# A Design of a Small-Aperture Low-Profile Omnidirectional Conformal Antenna

**DOI:** 10.3390/mi16020217

**Published:** 2025-02-14

**Authors:** Jieying Bai, Xi Li, Ziyu Zhang, Junjun Wu, Lin Yang

**Affiliations:** 1 National Key Laboratory of Antennas and Microwave Technology, Xidian University, Xi’an 710071, China; jieyingbai@stu.xidian.edu.cn (J.B.); 23021211799@stu.xidian.edu.cn (Z.Z.); lyang@mail.xidian.edu.cn (L.Y.); 2 Chinese Flight Test Establishment, Xi’an 710089, China; wu18792802634@outlook.com

**Keywords:** conformal antenna, low profile, omnidirectional, small aperture

## Abstract

In this article, a small-aperture, low-profile, and omnidirectional conformal antenna is proposed which can be utilized on space-limited equipment platforms such as airplanes, ships, and vehicles. The antenna consists of an open metal cavity, a discone antenna, a parasitic structure, and a radome. The small aperture and low-profile design of the metal cavity result in a rapid narrowing of the bandwidth of the discone antenna. Therefore, we introduce a parasitic structure that not only enlarges the impedance bandwidth by adding a resonant point, but can also be used to adjust the unroundness of the horizontal pattern. Meanwhile, the conformal design of the antenna with four surfaces of different curvatures is presented. The simulation and testing results demonstrate that the antenna can achieve a VSWR of less than 2 within a bandwidth of 1.95–2.62 GHz (29.3%), with a minimum aperture of 0.43 omnidirectional radiation pattern, with a gain exceeding −2.2 dBi in the azimuthal plane. This antenna offers the advantages of a small aperture, low profile, and conformal capability. Furthermore, the resonances of high and low frequencies can be adjusted through two different structures, enhancing the flexibility of antenna design.

## 1. Introduction

Antennas are increasingly used in a variety of scenarios, and in some curved surfaces, antennas are required to not only conformal to the surface but also ensure performance. Microstrip antennas are widely used in various conformal devices, such as microstrip wearable antennas and microstrip airborne vehicle antennas, because it is easy for them to conform to curved carriers. A multiband microstrip wearable antenna capable of achieving consistent characteristics under multiple curved surfaces was designed in [1]; an airborne microstrip antenna was designed in [2]; and airborne antennas capable of conforming to the surface of an airplane were designed in [3,4,5]. But the narrower bandwidth limits the application range of microstrip conformal antennas, and the bandwidth of conventional microstrip antennas is only 10%. Meanwhile, with the wide application of array antennas, the conformal design of array antennas is becoming more and more popular, and since the feasibility of the conformal of transmitter array antennas was proved in [6], the conformal design of transmitter array antennas has developed rapidly. In [7], an airborne tightly coupled conformal transmitter array for E-band applications is designed, and in [8], a single-fed multibeam conformal transmitter array capable of realizing phase and amplitude modulation is introduced. In [9], an optically transparent conformal reflector array is designed, and the designed antenna is capable of realizing 0° normal incidence efficient backscatter enhancement and a ±45° multi-angle backscatter enhancement in the X-band. With the rise in Metasurface antenna design, the conformal design of Metasurface array antennas has been mentioned more and more, and a conformal array design method for rectangular waveguide-fed metasurfaces is proposed in [10]. Reference [11] addressed the design of a Metasurface antenna for calculating the angle of arrival (AoA) of electromagnetic waves, which can be better applied to automobiles, aircrafts and missiles. However, these array antennas have the disadvantages of complex design and high machining accuracy. Slotted antennas are also able to realize conformal design on arbitrary curved surfaces [12,13,14,15,16,17], and are often grouped into arrays to realize specific functions, but slotted slit antennas have the same narrow bandwidth as microstrip antennas, which limits the range of applications.

Currently, commonly used airborne and vehicle-mounted omnidirectional antennas, in addition to the above-mentioned microstrip antennas and three kinds of array antennas, also have monopoles protruding from the surface of the equipment [18]. Airborne monopole antennas are evolving towards low-profile designs [19,20], and external omnidirectional antennas can also be modified into wire-frame structures to reduce aerodynamic interference [21]. However, the aircraft’s aerodynamics are still affected, leading to instability during rapid flight. The discone antenna with a metal back cavity designed in this paper can realize broadband omnidirectional radiation without affecting the aerodynamic performance, and has the advantages of simple structure and easy processing.

At present, all the equipment is developing in the direction of miniaturization to improve the utilization of equipment space. Antenna miniaturization research is also a hot topic in antenna development. Traditional antenna miniaturization methods include increasing the dielectric constant of the microstrip antenna, using meander or a folded curved structure, increasing the slit or shorting pins, and so on. Increasing the dielectric constant of the substrate of the microstrip antenna can reduce the antenna size and realize the miniaturization of the antenna [22,23]; the use of a meandering curved structure can increase the current path in the antenna design to reduce the antenna size to realize the miniaturization of the antenna [24,25]; and adding gaps and short-circuiting pins to the antenna structure can increase the equivalent capacitance or inductance of the antenna to realize the miniaturization of the antenna. The research on miniaturization in recent years has provided richer solutions. Metasurface antenna technology can realize the low profile of the antenna, and replace the dielectric impedance matching layer of the tightly coupled array antenna with the equivalent impedance matching layer of the Metasurface antenna to reduce the profile height of the antenna [26]. Thanks to the development of 3D technology, 3D stereoscopic and folding structure design will be the platform for the meander and folding curvature structure to expand into the space, which will further increase the flexibility of the design [27]. With the development of artificial intelligence, machine learning or genetic algorithms can be applied to automatically optimize the structure of the antenna to achieve miniaturization of the antenna design [28].

Q-factor can be used to measure the bandwidth limitation, energy storage characteristics and matching difficulty of the antenna, which is an important metric in the antenna design process. The relationship between the gain of a vertically polarized omnidirectional antenna and its size and quality factor (Q-factor) under ideal conditions is discussed in [29,30] by means of spherical wave function. The effect of antenna size on antenna Q-factor is analyzed in [31]. The relationship between planar aperture antenna and Q-factor is discussed in [32]. In [33], the optimal design of a small antenna for maximizing the maximum values of gain and quality factor ratio is presented. The inverse relationship between the impedance bandwidth of the antenna and the quality factor Q is derived in [34], and several commonly used antennas are listed to prove it. The antenna designed in this paper seeks both bandwidth and omnidirectional gain in the horizontal plane within a cavity of a certain aperture and profile height.

In this paper, a miniaturized antenna is designed by using gap and shorting pin loading inside the conformal cavity. In [35], an omnidirectional antenna with a circular aperture was designed, capable of achieving a VSWR < 2 within the frequency range of 3.4–6.8 GHz. However, this antenna features a relatively large aperture and a high profile. In [36], the authors broadened the antenna’s bandwidth by modifying the generator line of the radiation cone to a curve, loading metal shorting posts, and adding a metal ring at the top. However, this antenna still retained a larger aperture. The author achieved high-gain omnidirectional radiation by employing four discone antennas within a metal cavity in [37]. Nevertheless, this antenna design required four feed connectors, resulting in a relatively complex structure and a large aperture.

This paper presents an omnidirectional conformal antenna design that features a small aperture, low profile, and high gain. Reducing the profile height of the discone antenna and metal cavity size can result in a narrow impedance bandwidth [38]. In this paper, the impedance bandwidth is broadened by adding symmetric parasitic structures around the discone antenna. Constructed entirely of metal, except for the radome, this antenna offers the advantages of stability, reliability, and low loss. It can be seamlessly embedded into equipment surfaces with varying curvatures, without compromising its radiation performance.

## 2. Antenna Design and Analysis

### 2.1. Antenna Design

**Figure 1:**
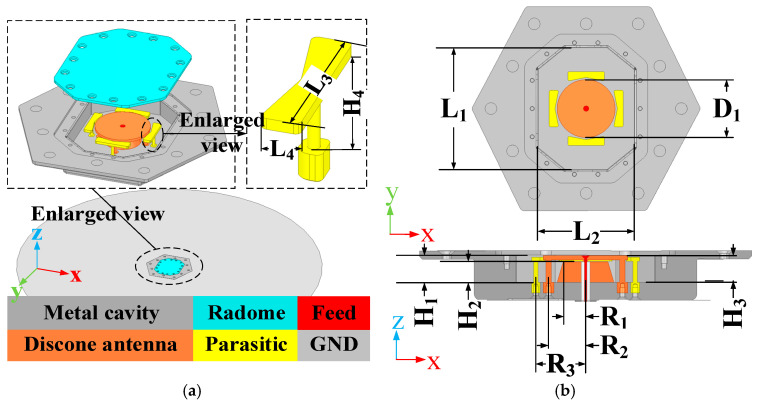
Antenna structure and dimensions: (**a**) installation and outline schematic; (**b**) top view and cross-sectional view.

Figure 1 presents the structure and dimensions of the antenna. The antenna was designed and simulated on a ground plane with a diameter of 1 m, achieving flush-mounting with the ground plane. The antenna is enclosed by an aluminum metal back cavity, with the radome covering the top opening of the cavity and conforming to the upper surface of the metal cavity. The radome is a single-layer pure dielectric sheet made of PEEK ($\varepsilon_{r}=3.3;\tan\delta =0.0035$). PEEK material has low losses and excellent mechanical properties to withstand the stresses generated by the rapid movement of mobile devices, such as airplanes. The shape and electromagnetic properties of the radome are set during the simulation phase. The radome serves not only to cover the top of the metal cavity, ensuring a conformal shape with the equipment’s surface, but also to incorporate a rubber ring that guarantees airtight sealing during assembly. This design prevents water ingress, safeguarding the antenna’s normal operation and prolonging its service life.

**Figure 2:**
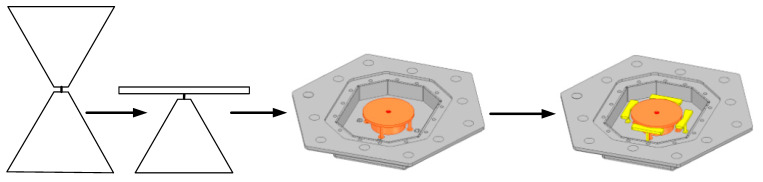
Diagram of the antenna design process.

**Figure 3:**
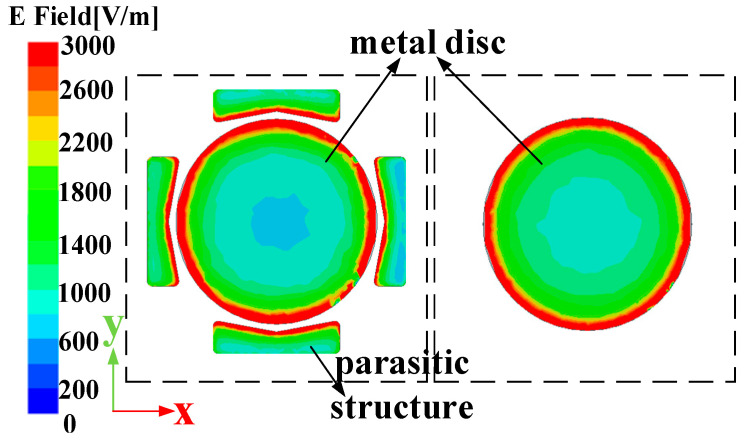
Comparison of electric field distribution on the antenna with and without parasitic structures.

**Figure 4:**
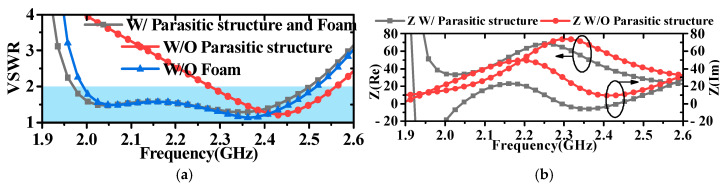
Comparison of VSWR and impedance with and without parasitic structures: (**a**) VSWR; (**b**) impedance.

**Figure 5:**
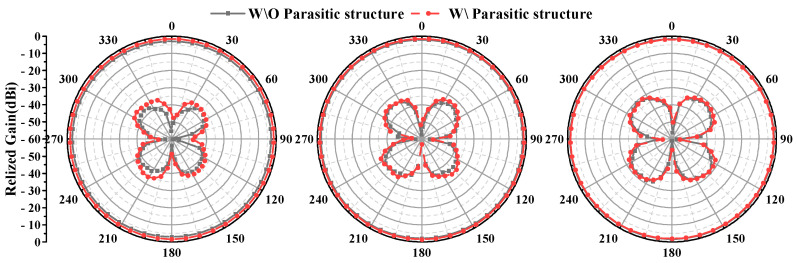
Diagrams of horizontal gain with or without parasitic structures.

**Figure 6:**
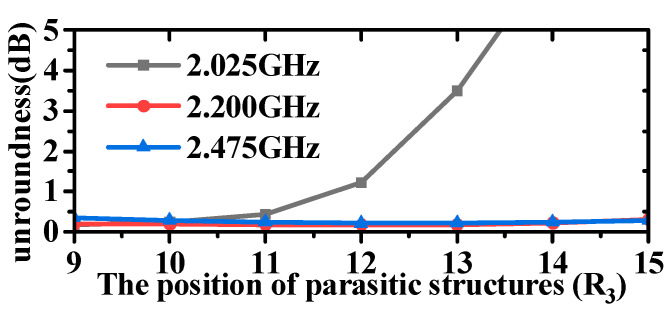
Effect of parasitic structure position on unroundness.

The design process of the antenna is shown in Figure 2. The discone antenna can be regarded as an evolution from the biconical antenna, significantly reducing the antenna’s profile height [39,40]. The discone antenna is positioned at the centre inside the metal cavity, with its top metal disc connected to the cavity through four metal shorting posts. These four posts are evenly distributed around the metal disc. Unlike common discone antennas, this paper introduces four parasitic structures around the discone antenna to increase the bandwidth of the antenna [13,41], but unlike [13,41], in this paper, the parasitic structures are added inside the metal cavity, and the dielectric support is replaced by a metal column to support it, and it is designed as a bowtie structure, as shown in Figure 1a, to optimize the coupling with the discone antenna. The electric distribution on the antenna is shown in Figure 3, illustrating that the parasitic structures generate a coupled electric field. The introduction of parasitic structures can broaden the bandwidth, which can be analyzed from the perspectives of impedance matching and the principles of loop antennas. From the impedance perspective, as illustrated in Figure 4b, it is evident that the low-frequency input impedance (Zin = Re + jIm) does not match the 50-ohm coaxial feed prior to loading. The real part of the impedance (Re) is lower than 50 Ω at low frequencies and higher than 50 Ω at high frequencies. With the addition of the parasitic structure, the Re increases at the low frequencies and decreases at the high frequencies, but the overall change tends to be around 50 Ω and is much smoother. The imaginary part (Im) is large and inductive. The gap between the discone antenna and the parasitic structure can be viewed as adding a coupling capacitance that can cancels out the low-frequency inductance. Therefore, by incorporating the parasitic structure, low-frequency impedance matching can be achieved. From the perspective of loop antennas, the parasitic structures evenly distributed around the discone antenna can be regarded as loop antennas. According to the principle of loop antennas [13,41,42], loop antennas can excite multiple modes, with the dominant mode being the TM11 mode. Based on empirical equations, the operating frequencies of different modes of the loop antenna can be calculated by the following formula:

(1) $$f_{0}=\frac{ck}{2\pi \sqrt{\varepsilon_{eff}}}$$

(2) $$k\approx \frac{2n}{\left(R+r\right)}$$

where *c* is the speed of light in air, *k* is the root of the characteristic equation for the operating mode, and $\varepsilon_{eff}$ is the equivalent dielectric constant, which in this case is air, so $\varepsilon_{eff}=1$. *R* and *r* are the outer and inner radii of the loop, respectively, $(R+r)\approx 2R_{3}$. Here, *n* represents *n* in the TM_nm_ mode. Since it is operating in the dominant mode, *n* = 1. Through calculation, the operating frequency of the loop antenna is obtained as 2.03 GHz. As shown in Figure 4a, the operating frequency of 2.03 GHz obtained by theoretical calculation is consistent with the increased resonance point at the low frequency in the simulation. A loop antenna generates an omnidirectional radiation field with horizontal polarization, whereas a discone antenna produces an omnidirectional radiation field with vertical polarization [13,38,43,44]. As shown in Figure 5, the introduction of parasitic structures degrades the antenna’s cross-polarization level, yet it remains above 30 dBi. In addition, as shown in Figure 6, the position of the parasitic structure (R_3_) can adjust the unroundness of the antenna. Unroundness represents the uniformity of radiation from an omnidirectional antenna. It can be calculated by the following formula.

(3) $$G_{unroundness}=G_{\max}-G_{\min}$$

where $G_{\max}$ and $G_{\min}$ represent the maximum and minimum value of the gain in the omnidirectional pattern, respectively.

As shown in Figure 1, the parasitic structures are connected to the metal cavity through metal pins. To ensure the stability of the antenna within the metal cavity, PMI foam is used to fill the gaps. It can be seen from Figure 4a that the foam has little influence on the VSWR of the antenna.

**Figure 7:**
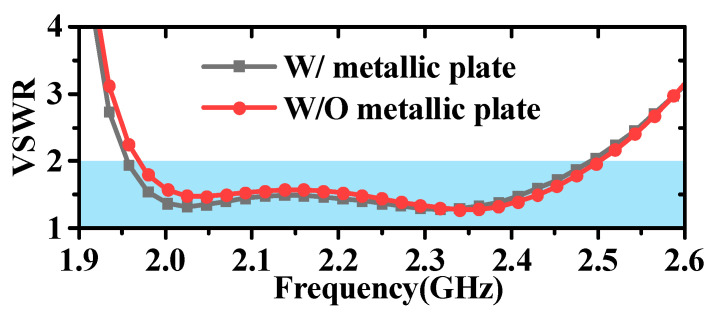
Diagram of VSWR with and without metallic plate.

**Figure 8:**
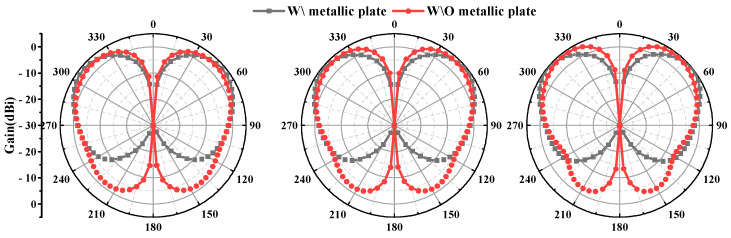
Diagram of radiation pattern with and without metallic plate.

**Figure 9:**
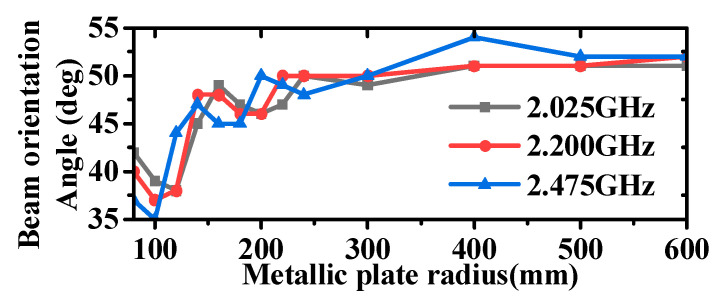
Diagram of the beam pointing angle as a function of the metallic plate radius.

**Figure 10:**
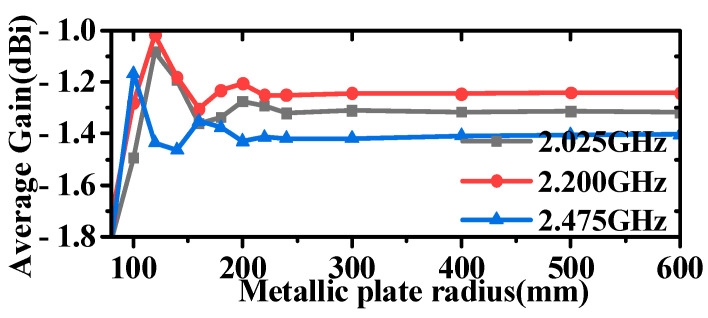
Diagram of the average gain as a function of the metallic plate radius.

Both the simulation and testing of the antenna were conducted on a metal disc with a diameter of 1 m. The impact of the metal disc on the antenna’s performance is also analyzed here, and the results are shown in Figure 7 and Figure 8. It can be observed that the inclusion or lack of the metallic plate has minimal impact on the antenna’s VSWR, with only slight effects at lower frequencies. However, the impact on the radiation pattern is significant. In the absence of the metal plate, the antenna exhibits increased backward radiation, a reduced angle of maximum gain, and a slight increase in gain in the horizontal plane. Considering the practical applications of antennas, which are typically mounted on large platforms such as airplanes, ships, and vehicles, the inclusion of a metal plate is a crucial consideration. Additionally, the antenna measurements reported in references [35,37,45,46] were all conducted on relatively large ground planes compared to the antenna aperture size. Further analysis of the specific impact of metal plate size on antenna radiation is illustrated in Figure 9 and Figure 10; as the size of the metal plate increases, the beam-pointing angle initially decreases, then increases, and finally stabilizes. Correspondingly, the gain in the horizontal plane first increases, then decreases, and eventually plateaus. This phenomenon can be attributed to the fact that as the metal plate size decreases, the ground plane’s diffraction of incident radiation becomes more pronounced. However, it can be seen from Figure 9 and Figure 10 that when the radius of the metal floor is larger than 400 mm, the beam-pointing angle and the average gain in the horizontal plane hardly change with the size of the radius of the metal plate. Considering that the antenna installation platform is an airplane, a ship, etc., the surface size of the equipment will be much larger than the 800 mm metal plate, so the influence of the metal plate on the antenna performance can be ignored.

**Figure 11:**
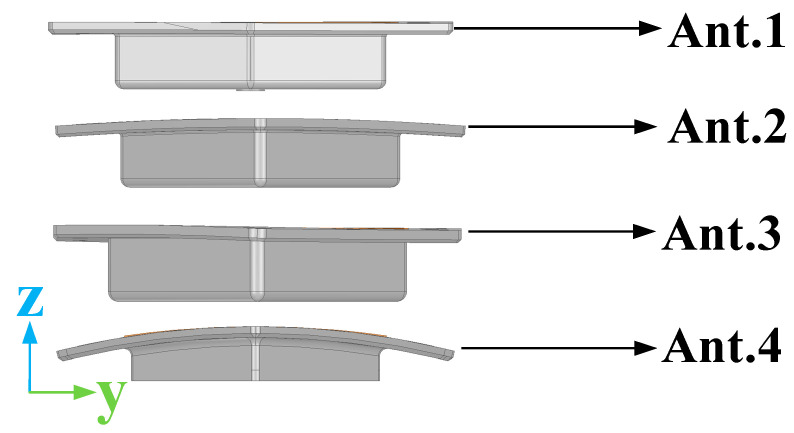
Left view of four antennas.

The designed antenna can be adjusted to conform to surfaces with varying curvatures. Specifically, simulations were conducted for antennas conforming to four types of surfaces with distinct curvatures, as illustrated in Figure 11. In this paper, Ant. 1 was primarily utilized to investigate the characteristics of the antenna.

### 2.2. Matching Analysis

**Figure 12:**
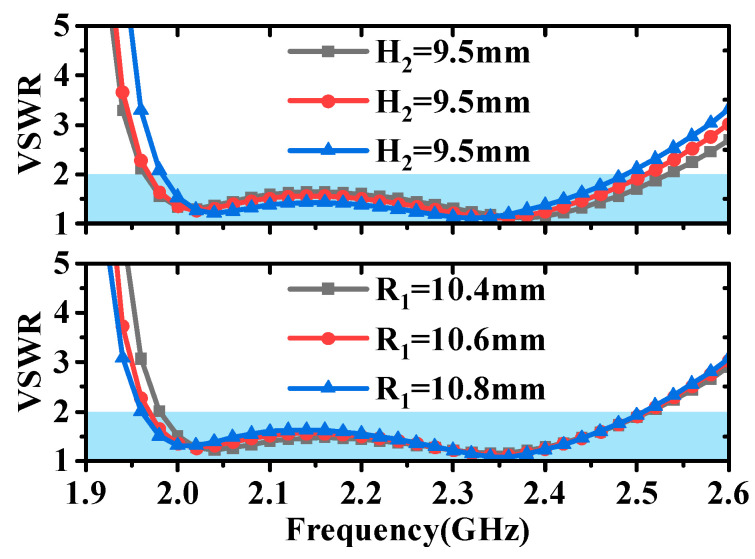
Impact of metal cone height (H_2_) and radius (R_1_) on antenna matching.

**Figure 13:**
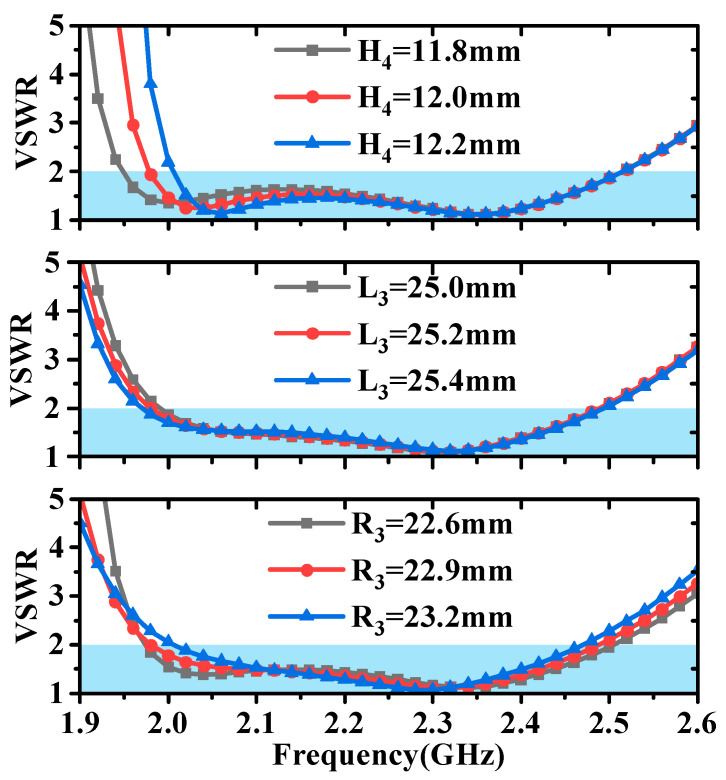
Impact of height (H_4_), length (L_3_), and position of parasitic structures (R_3_) on antenna matching.

From Figure 12, it can be observed that the height of the metal cone (H_2_) has an impact on matching across the entire frequency range, while the upper radius of the metal cone (R_1_) primarily affects matching at low frequencies. From Figure 13, it is evident that the height (H_4_) and length (L_3_) of the parasitic structure mainly influence matching at low frequencies. The position of the parasitic structure (R_3_) not only has a significant effect at low frequencies but also exerts a slight influence at high frequencies. By analyzing the impact of these parameters on antenna matching, we can see that the introduction of the parasitic structure allows for independent control of the high- and low-frequency VSWR of the antenna, thereby enhancing the flexibility of the antenna design.

Full wave simulation software (Ansoft HFSS 2022R1) was utilized to simulate and optimize the antenna, and the optimized parameters are presented in Table 1. Ansoft HFSS 2022R1 is based on the Finite Element Method (FEM) and electromagnetic field theory to simulate the radiated fields of the model, which are adaptively dissected into a grid by constructing the physical structure of the model (as shown in Figure 1), the material, the port excitation, and the frequency band of operation. The software converts Maxwell’s equations into matrix equations and performs iterative calculations to solve for the distribution of electric and magnetic fields. The adaptive mesh splitting is based on the results of the solution, and the mesh splitting will be denser in the smaller part of the structure to ensure the accuracy of the solution.

### 2.3. Quality Factor Analysis

Q-factor is an important parameter for antenna design and evaluation. *Q_C__hu_* is used as a reference for the bandwidth limit value of electrically small antennas in most cases. The antenna designed in this paper is not strictly an electrically small antenna, but it belongs to the compact antenna category, which faces the same challenge as electrically small antennas to improve the bandwidth and efficiency with a limited size during the design process. Therefore, *Q_C__hu_* can still provide some reference for the optimized design in this paper. *Q_Chu_* can be calculated using the following equation:

(4) $$Q_{Chu}=\frac{1}{{\left(ka\right)}^{3}}+\frac{1}{ka}$$

where $k=\frac{2\pi }{\lambda }$, λ is the operating wavelength, and a is the equivalent radius of the antenna. Calculating the *Q_Chu_* value designed in this paper, *Q_Chu_* is obtained to be about 0.58 at *a* = 44 mm, *k* = 0.047.

The actual Q-factor of the antenna can be calculated according to the following equation:

(5) $$Q=\frac{f_{0}}{\Delta f}$$

where $f_{0}$ is the resonant frequency and Δf is the operating bandwidth. The actual Q-factor calculated for this paper is about 3.41. Calculating the Q-factor of the antenna without a parasitic structure according to the above equation gives a factor of about 9.7. This also proves that the Q-factor decreases after loading the parasitic structure, achieving the broadening of the antenna bandwidth [34].

To analyze the relationship between the horizontal plane gain and Q-factor of the omnidirectional antenna designed in this paper, the relationship between the antenna gain and Q-factor is given in [29]; when 2πa/λ≈2;N=1, the omni-directional horizontal gain under ideal conditions is about 1.5 (1.76 dBi), and the gain obtained from the processing test of this paper is 1.05 dBi, which is not much different from the ideal gain.

A comprehensive analysis of the Q-factor, bandwidth, and gain of the compact antenna shows that the Q-factor is approximately inversely related to the antenna bandwidth and gain [29,30,33,34].

From the antenna evolution process, the discone antenna in [33] operates at 3.1–10.6 GHz with a low Q-factor of 0.913, but when the discone antenna is placed inside a compact metal cavity, this leads to a rapid increase in the Q-factor and narrowing of the bandwidth [26,37], which is 9.7 in this paper. In this paper, we reduce the antenna’s Q-factor (3.41) by adding a parasitic structure inside the metal cavity, which in turn broadens the bandwidth of the antenna.

## 3. Measured Result

In this paper, four antennas with different surface curvatures were designed. Ant. 4 was only simulated in Ansoft HFSS 2022R1. The antenna’s VSWR was measured in an anechoic chamber using a vector network analyzer (Rohde & Schwarz ZVB20, Rohde & Schwarz, Beijing, China). Specifically, the test procedure is as follows:

(1) Open the vector network analyzer and use the supporting calibration parts to complete the calibration.
(2) Set the working frequency range of the antenna to be tested (the antenna in this paper); generally the set range will be slightly wider than the actual range.
(3) Measure VSWR or S parameters.
(4) Export the results and complete the measurement.

**Figure 14:**
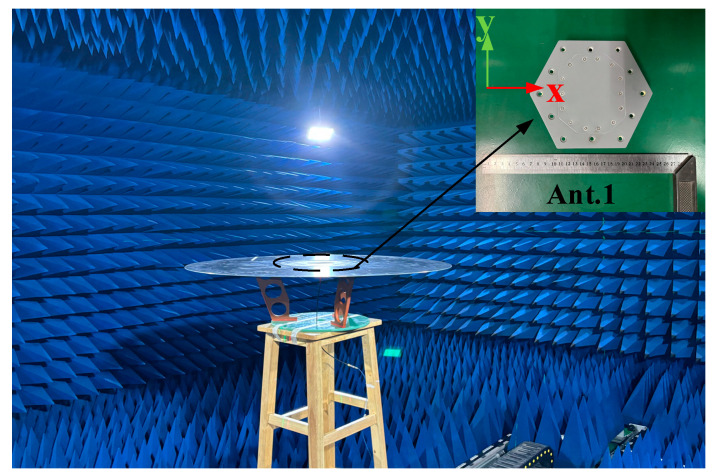
Diagram of the antenna test environment.

**Figure 15:**
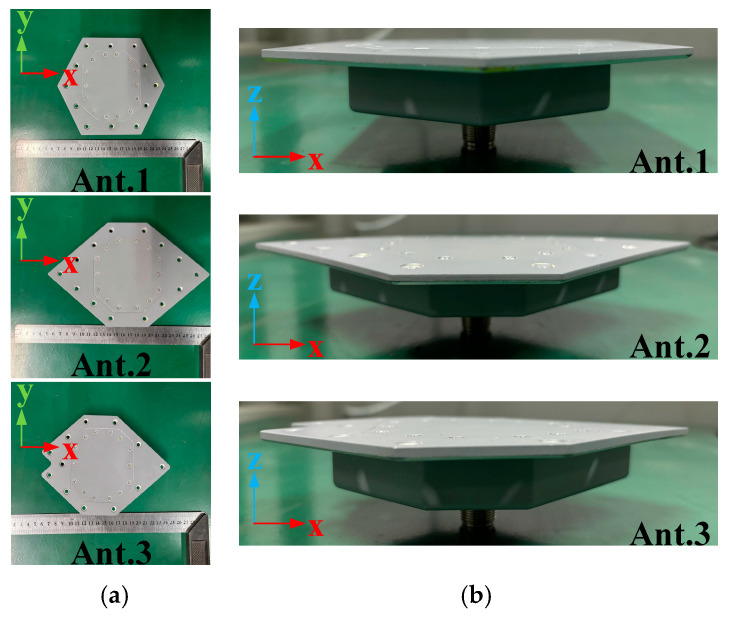
Diagrams of antennas on three different curvature surfaces: (**a**) top view; (**b**) front view.

**Figure 16:**
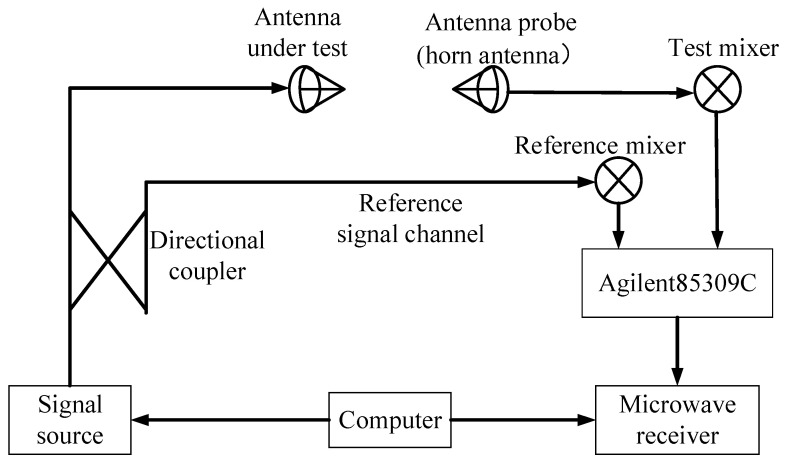
Diagram of the antenna test system.

As shown in Figure 14 and Figure 15, Ant.1, Ant.2, and Ant.3 were fabricated and subsequently measured in the far field using a ridged horn antenna (1–18 GHz) as the receiving antenna in a microwave anechoic chamber. Considering the cost of the experiment and the weight of the antenna, aluminum was used (except for the radome). The antenna was machined so that it could be processed in the following parts: the metal cavity with cone, the metal disc, the top of the parasitic structure, eight metal pins, and a dielectric radome. After machining, the assembly was completed using screws according to Figure 1. The gain of the antennas was measured using a comparison method. The principles of the test system for the radiation pattern and gain are shown in Figure 16, and the specific measurement steps are as follows:

(1) Prepare the work, making sure that each device of the measurement system is connected correctly and in normal working condition.
(2) Move the turntable to ensure the distance between the probe (1–18 GHz working ridge horn) in the far-field region of the antenna under test (AUT, the antenna in this paper); the minimum required distance is 2D2/λ, where D is the maximum size of the antenna and λ is the working wavelength. The distance between the turntable and the probe in the actual test is 10 m.
(3) Connect the test system with the probe (1–18 GHz working ridge horn) and connect the test system with the AUT.
(4) Place the AUT on a rotation table with its bottom surface parallel to the horizontal plane. Adjust the height of the turntable using a laser aligner to ensure that the phase centre of the probe and the AUT are aligned.
(5) Set the measurement range according to the working range of the AUT, and ensure that the probe and the AUT polarization are consistent.
(6) Turn the rotary table in a circle (resolution: 0.05°) and complete the measurement of the AUT.
(7) Replace the AUT with the standard gain horn antenna, and follow the above process again to complete the measurement of the standard gain horn antenna.
(8) The measured value is the power value, found through the known gain of the standard gain horn antenna and when using the following formula to calculate the gain of the AUT (P is the measured power level value).

(6) $$Gain_{AUT}=P_{AUT}-P_{Horn}+Gain_{Horn}$$

**Figure 17:**
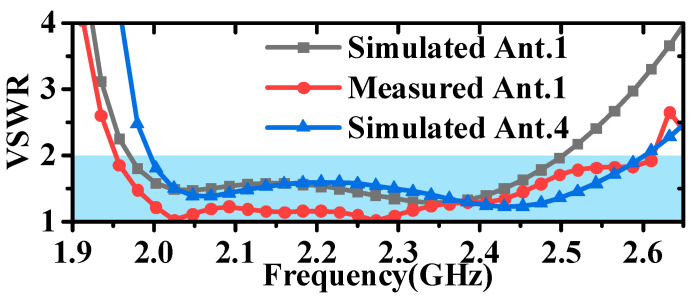
Diagram of simulated and measured VSWR for the antenna.

**Figure 18:**
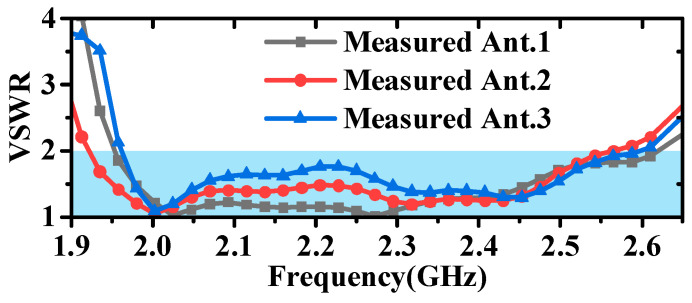
Diagram of measured VSWR for antennas.

**Figure 19:**
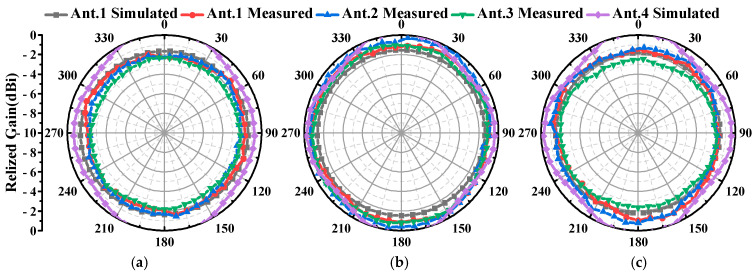
Simulation and measured radiation patterns (xoy plane) and gain: (**a**) 2.025 GHz; (**b**) 2.2 GHz; (**c**) 2.475 GHz.

**Figure 20:**
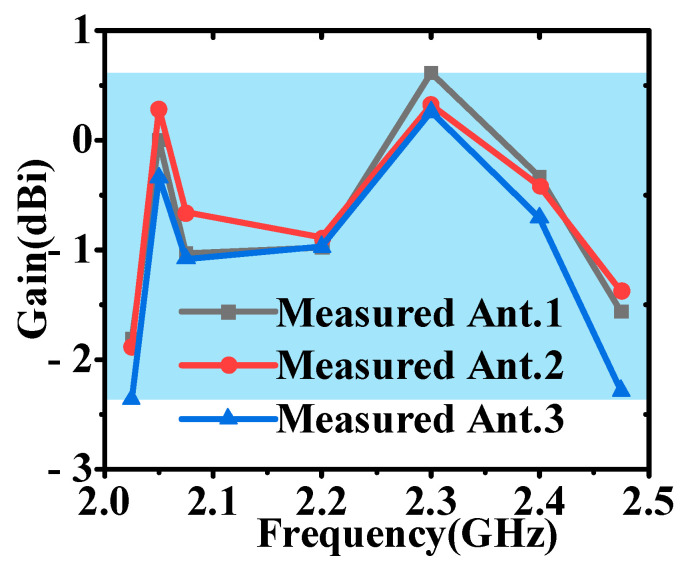
The measured horizontal average gain of Ants. 1, 2, and 3.

As demonstrated by the simulation and measurement results presented in Figure 17 and Figure 18, these antennas achieved a VSWR of less than 1.98 within the 2–2.5 GHz frequency band. As shown in Figure 19 and Figure 20, the horizontal gain of the three antennas was above −2.2 dBi and the unroundness was less than 1 dB. Additionally, as the curvature increased, the measured VSWR deteriorated, but the gain increased. It is also noteworthy that Ant. 4, which has the largest conformal surface curvature, exhibited a pattern gain in the 0° and 180° directions that was significantly higher than in the 90° and 270° directions. This configuration experienced a significant deterioration in unroundness, but still less than 3 dB. When compared to simulation results, there is a decrease in minimum gain and an increase in pattern fluctuation, which could potentially be attributed to antenna assembly and measurement errors.

## 4. Discussion

Table 2 presents a comparison of the key performance metrics between the antenna simulated in this paper and other similar antennas. In comparison to the antenna in [26], the antenna presented in this paper is easier to manufacture and can achieve omnidirectional radiation within a lower profile and smaller aperture. Although the gain in the horizontal plane is not explicitly given in [26], it can be roughly estimated from the provided figure to be approximately −2 dBi. Unlike the design in [37], which utilizes an array of four antennas, this paper employs only a single antenna. Additionally, although the antenna in [37] is conformal to a curved surface, the stability of the antenna’s radiation performance when the surface curvature changes has not been investigated. In contrast to ANTENNA II in [45] and the design in [46], this paper attains a higher gain with a smaller aperture. Overall, this paper introduces the design of a high-gain omnidirectional radiation antenna within a small-aperture, low-profile cavity.

## 5. Conclusions

In this article, we propose an omnidirectional antenna designed for flush-mounting with the surface of a device. This antenna is capable of achieving omnidirectional radiation with a horizontal plane gain exceeding −2.2 dBi within a small-aperture and low-profile cavity. By incorporating parasitic structures around the discone antenna, the antenna achieves a minimum aperture of 0.43 $\lambda_{\max}$ and a profile height of 0.084 $\lambda_{\max}$ Simulation and measurement results further indicate that this antenna can adapt to surfaces with different curvatures, meeting the miniaturization and conformal requirements for airborne, shipborne, and vehicle-mounted applications.

## Figures and Tables

**Table 1:** Parameters of the optimized antenna.

Parameter	L_1_	L_2_	L_3_	L_4_	H_1_	H_2_
Value (mm)	84	66	25.2	6.3	12.9	9.7
Parameter	H_3_	H_4_	D_1_	R_1_	R_2_	R_3_
Value (mm)	12.7	12	40	10.6	17.5	23.5

H_1_: The height of the antenna cavity; H_3_: the distance from the metal disc to the bottom of the cavity.

**Table 2:** Comparison of the proposed antenna with other similar antennas.

Ref.	Conformal Surface	BW (GHz)	$\mathrm{Aperture}\;\mathrm{Size}\;(\lambda_{\max}$)	$\mathrm{Height}\;(\lambda_{\max}$)	Az. Gain (Phi = 0°)
[26]	Plane surface	0.96–1.22 GHz < 2 (23.85%)	$280\;\mathrm{mm}\;(0.896\;\lambda_{\max}$)	$43\;\mathrm{mm}\;(0.137\;\lambda_{\max}$)	about −4 dBi
[37]	Curved surface	0.96–1.215 GHz < 2 (23%)	$215.9\;\mathrm{mm}\;(0.69\;\lambda_{\max}$)	$58.6\;\mathrm{mm}\;(0.18\;\lambda_{\max}$)	−1~−4.4 dBi
[45]	Plane surface	0.8–2.5 GHz < 1.93 (103%)	$280\;\mathrm{mm}\;(0.74\;\lambda_{\max}$)	$26\;\mathrm{mm}\;(0.069\;\lambda_{\max}$)	−4~−7 dBi
[46]	Plane surface	4.7–7.6 GHz < 2 (47%)	$74\;\mathrm{mm}\;(1.15\;\lambda_{\max}$)	$8\;\mathrm{mm}\;(0.125\;\lambda_{\max}$)	−10~−20 dBi
This work	Curved surface	1.95–2.62 GHz < 2 (29.3%)	$66\;\mathrm{mm}\;(0.43\;\lambda_{\max}$)	$12.9\;\mathrm{mm}\;(0.084\;\lambda_{\max}$)	1.05~−2.3 dBi

$\lambda_{\max}$: Free-space wavelength corresponding to the lowest operating frequency.

## Data Availability

The datasets presented in this article are not readily available be cause the data are part of an ongoing study. Requests to access the datasets should be directed to jieyingbai@stu.xidian.edu.cn.
